# Processing, Bioaccessibility, Predicted Bioavailability, Gut Microbiome Interactions and Potential Food Applications of *Vernonia amygdalina* (Bitter Leaf): Bridging the Evidence-to-Practice Gap

**DOI:** 10.3390/foods15172962

**Published:** 2026-08-23

**Authors:** Khavhatondwi Rinah Mofokeng, Rhulani Caswell Chauke

**Affiliations:** 1 Department of Life and Consumer Sciences, University of South Africa, Private Bag X6, Florida 1710, South Africa; 2 Department of Nutrition, Faculty of Health Sciences, University of Venda, Private Bag X5050, Thohoyandou 0950, South Africa

**Keywords:** *Vernonia amygdalina*, food processing, nutrient retention, bioaccessibility, predicted bioavailability, lactic acid fermentation, gut microbiome, indigenous leafy vegetables

## Abstract

*Vernonia amygdalina* Del. (bitter leaf) is a nutrient-rich indigenous leafy vegetable, but most research has examined raw material or laboratory extracts rather than the vegetable as consumed. This structured narrative review evaluates how household and food-processing methods affect nutrient retention, anti-nutritional factors, and bioaccessibility and distinguishes measured species-specific findings from extrapolated evidence. Boiling and aqueous debittering can reduce heat-sensitive nutrients, minerals, and anti-nutritional factors, but the heterogeneous primary studies do not permit a single quantitative estimate of these effects. Lactic acid fermentation is a potential research strategy: proposed reductions in anti-nutritional factors and bitterness are extrapolated from other fermented plant foods because no species-specific fermentation study was identified. Similarly, proposed gut-microbiome effects are inferred from general dietary-fibre and isolated-polyphenol literature, not from *V. amygdalina* feeding studies. No human study has directly measured nutrient absorption, systemic bioavailability, or clinical effects after consumption of processed bitter leaf. Hence, phytate-to-mineral ratios are interpreted only as indicators of predicted absorption, and proposed food formats and development pathways are identified as author-generated concepts rather than validated products. The review identifies priorities for species-specific processing studies, standardised in vitro digestion, product safety and acceptability testing, and ultimately human absorption and efficacy research.

## 1. Introduction

Few African indigenous leafy vegetables have been studied as extensively as *Vernonia amygdalina* Del. (bitter leaf). The leaves contain protein, fibre, carotenoids, minerals and bioactive compounds, including sesquiterpene lactones, flavonoids, saponins and phenolic acids [1,2,3]. Extract-based and preclinical studies of *V. amygdalina* have reported antidiabetic, anti-inflammatory and anticancer activities [4,5,6,7,8]. A broader review of sesquiterpene lactones provides mechanistic context for anti-inflammatory activity but is not species-specific evidence for *V. amygdalina* [9]. These findings support biological plausibility but do not demonstrate therapeutic effects of bitter leaf consumed as food. Most pharmacological studies used freeze-dried powders, isolated compounds, or aqueous or organic-solvent extracts at exposures that may differ significantly from household meals. In many African communities, bitter leaves are washed, squeezed, soaked, boiled, or otherwise debittered before consumption; these practices can change nutrient and phytochemical composition through leaching, heat, oxidation, and enzymatic activity. The nutritional value of the consumed vegetable must therefore be distinguished from pharmacological activity observed in laboratory preparations.

This distinction determines how the evidence is interpreted. In this review, “functional potential” refers only to proposed benefits from composition, mechanistic studies or preclinical evidence; it does not indicate an authorised health claim or a demonstrated human effect. In the absence of human absorption and intervention studies, *V. amygdalina* is described primarily as a nutrient-rich indigenous leafy vegetable with unconfirmed functional potential.

The importance of *V. amygdalina* extends beyond scientific interest. African populations continue to experience hunger, micronutrient deficiencies, and diet-related non-communicable diseases [10,11,12]. Bitter leaf is already cultivated and consumed in several settings, but its contribution to sustainable nutrition depends on locally demonstrated agronomic performance, safe processing, affordability, and consumer acceptance. This review follows the food from processing to digestion and potential absorption. It evaluates nutrient retention and anti-nutritional factors, distinguishes bioaccessibility from predicted and measured bioavailability, examines proposed microbiome interactions, assesses potential food applications and regulatory categories, and identifies the evidence required before nutritional or health claims can be justified.

## 2. Methods

### 2.1. Literature Search Strategy

Relevant literature was identified through structured searches of Scopus, Web of Science Core Collection, PubMed, ScienceDirect, Google Scholar, African Journals Online and Sabinet. Reference lists of relevant reviews were also examined, and official FAO, WHO, South African government, and regulatory sources were consulted. The core Boolean structure combined (“*Vernonia amygdalina*” OR “bitter leaf” OR selected common names) with terms for processing, boiling, blanching, drying, fermentation, nutrient composition, anti-nutritional factors, bioaccessibility, bioavailability, gut microbiota, product development, sensory evaluation, policy and regulation. The core concepts were adapted to the field codes and syntax of each database; corrected representative strings are provided in Table A1 (Appendix A). Because searches were conducted iteratively and the original search dates were not recorded for every database, this review does not claim the reproducibility or completeness of a systematic review.

The search covered publications from January 1990 to July 2026, with no language restriction at initial consideration. Eligible sources included species-specific primary studies relevant to food processing, composition, or digestion; reviews used for context; mechanistic studies used only for explicitly labelled extrapolation; and official policy or regulatory documents. Laboratory solvent-extract studies were included only when needed to explain biol*ogi*cal plausibility and were not used to assess effects of the consumed food. Sources were excluded when the plant identity was not confirmed, the processing method was not identifiable, the full text was unavailable, or the work had no relevance to the review questions. Only species-specific primary studies could support a measured processing statement. Studies with unclear moisture basis or insufficiently described conditions were retained for directional discussion but not for cross-study numerical comparison.

### 2.2. Study Selection

The lead author conducted title and abstract screening and full-text eligibility assessment; screening was not independently duplicated. The second author reviewed the final included source set and the interpretation of major evidence statements. Differences in interpretation were resolved through discussion. Duplicate records were identified during manual reference-library review using title, author, year, and DOI where available; however, duplicate-removal counts were not preserved. Similarly, the numbers identified, screened, and excluded at each stage and full-text exclusion reasons were not recorded in the historical working files and cannot be reconstructed reliably. No PRISMA flow diagram is therefore presented.

This review does not claim full coverage. Statements that evidence was “not identified” refer to the searches described here and do not prove that no study exists. The iterative search, absence of independent duplicate screening, lack of preserved flow counts and reliance on one primary screener may have favoured familiar or accessible sources. Strong comparative claims were therefore avoided unless supported by directly relevant species-specific evidence, and extrapolations are labelled as hypotheses or research priorities.

### 2.3. Terminology

To ensure consistency, four key terms are used with specific meanings throughout this review because they are often used interchangeably in literature.

- Bioaccessibility refers to the proportion of a nutrient or bioactive compound released from the food during digestion and made available for absorption. It is measured using in vitro digestion models and does not indicate whether absorption occurs.
- Absorption is the movement of a nutrient or compound across the intestinal wall into the body.
- Bioavailability is the fraction of an ingested nutrient or bioactive compound that reaches systemic circulation and is available for physiological use. It may be evaluated in animal or human in vivo studies, but human studies are required to establish human bioavailability [13].
- Metabolite bioavailability refers to the fraction of metabolites formed after digestion, microbial transformation or host metabolism that reaches systemic circulation and becomes available for physiological use; it does not refer simply to the number of metabolites produced [13].

Throughout this review, outputs from in vitro digestion, compositional analysis or phytate-to-mineral ratios are described as bioaccessibility, composition or predicted absorption, as appropriate. They are not treated as measured human bioavailability. No human study identified here directly measured nutrient or phytochemical bioavailability from *V. amygdalina*.

### 2.4. Evidence Classification

The evidence classification in Table 1 was developed by the authors specifically for this narrative review; it was not adapted from and should not be interpreted as a validated evidence-certainty or risk of bias framework. Its purpose is to identify whether a statement derives from human, animal, species-specific laboratory, or extrapolated evidence. The lead author assigned the initial classes, and both authors reviewed the final assignments and resolved differences through discussion. Reporting quality was considered separately through plant authentication, moisture basis, processing detail, analytical method, replication, and variability. The classes guide permissible interpretation but do not rank study quality or establish certainty of evidence.

## 3. Botanical and Ethnobotanical Context

*Vernonia amygdalina* Del. is a perennial shrub of the Asteraceae that grows widely in tropical Africa, and its leaves are used as food and in traditional practices [1,2]. It is commonly propagated from stem cuttings and cultivated in several African settings, but agronomic requirements, time to harvest, and performance vary with environment and management. Claims that it universally requires minimal inputs, reaches harvest in 8–12 weeks, or constitutes a sustainable nutrient source are not supported consistently enough for generalisation. Its sustainability must be assessed in relation to local water, soil, production and supply-chain conditions. Local names and preparation practices vary across regions; verified Nigerian names include ewuro in Yoruba and onugbu in Igbo [1,2]. These practices matter because washing, squeezing, soaking, and heating can alter the composition of the consumed portion.

Moisture content is another important factor when interpreting nutritional data. Fresh bitter leaves have a high moisture content (on the order of 80% on a wet-weight basis) that falls to only a few percent after sun- or oven-drying [14]; the precise values depend on cultivar, maturity and drying conditions. As water is removed, the concentrations of protein, fibre and minerals appear much higher on a dry-weight basis than in fresh leaves. Therefore, comparing studies that report fresh, dried or cooked leaves without clearly stating the moisture basis can lead to misleading conclusions and overestimating the nutritional value of the leaves as they are normally consumed.

## 4. Effects of Processing on Nutritional Composition and Bioactive Retention

### 4.1. The Comparability of the Primary Literature

The species-specific processing literature is small and heterogeneous in relation to methodology. Studies differ in plant source, processing treatment, duration, water use, analytical methods, and whether results are reported on a fresh or dry weight basis. Moisture removal can increase the apparent concentration of retained constituents even when absolute amounts decrease. Table 2 therefore reports the treatment and outcomes of each source separately. Cross-study percentage ranges are not calculated because, unlike treatments, moisture bases are not directly comparable. Findings are interpreted only within the conditions reported by the original study and are unsuitable for single analysis, dietary modelling or product formulation.

### 4.2. Boiling and Thermal Processing

Household heating and aqueous debittering may change bitter-leaf composition through heat degradation, oxidation and leaching, but the primary studies do not support a universal percentage loss for a standard 15- or 30-min boiling treatment. Yakubu et al. examined soaking, blanching and abrasion treatments [15]; Tekou et al. compared culinary boiling approaches for phenolics, dietary fibre and related outcomes [16]; and Shokunbi et al. examined abrasion with and without salt rather than controlled boiling [18]. These treatments should not be combined into a single numerical range. The defensible conclusion is therefore qualitative: aqueous and thermal processing can reduce some measured constituents, while the magnitude depends on the treatment, analytical basis, and fate of the processing water.

Reductions in phytate, oxalate, vitamins or minerals should therefore be reported only for the treatment measured in the cited study. The available sources do not report that boiling for 15–30 min consistently reduces phytate by 30–60% or oxalate by 25–50%, nor do they report the effect of household boiling on sesquiterpene lactones. Species-specific experiments should report raw and processed values on a common moisture basis, water-to-leaf ratio, time, temperature, and whether processing water is consumed or discarded.

### 4.3. Sun-Drying and Oven-Drying

Drying extends shelf life and permits powder production, but the nutritional effect depends on pretreatment, temperature, duration, light exposure, and reporting basis. Aliero and Abdullahi compared solar, sun, and oven drying for proximate and mineral composition [14]; noticeable concentration increases after drying must be interpreted in relation to moisture loss. The included studies do not report 40–60 °C as a universally optimal range for retaining β-carotene or phenolics in *V. amygdalina*. General carotenoid-stability literature may inform hypotheses [17] but cannot define a species-specific optimum. Freeze-drying may preserve some heat-sensitive compounds in laboratory preparations, yet comparative species-specific evidence and economic feasibility data are insufficient for a general claim.

### 4.4. Fermentation

No published species-specific fermentation study was identified. Every proposed effect in this section is drawn from other fermented plant foods or general fermentation mechanisms and is not a demonstrated outcome in *Vernonia amygdalina*. Lactic acid bacteria can reduce phytate or modify phenolic compounds in some plant matrices [19], but the direction and magnitude depend on substrate, organism, oxygen, time, and temperature. Possible effects on bitterness, antioxidant measurements, oxalate, or consumer acceptance remain untested in bitter leaf. A recent comprehensive review reports antibacterial and antifungal activity for some bitter-leaf preparations or isolated compounds [1], but no cited study reports inhibition of fermentation-relevant *Lactobacillus*, *Leuconostoc* or *Pediococcus* in a bitter-leaf substrate. Fermentation should therefore be described as a research opportunity requiring starter-culture screening, kinetic measurement, safety assessment and sensory testing, not as an established improvement method.

### 4.5. Blanching and Combined Treatments

Blanching is a short heat treatment, but no universal bitter-leaf blanching protocol can be found from the studies included. Yakubu et al. included blanching among several processing treatments [15], whereas other sources used different aqueous or mechanical debittering methods. Effects must be attributed to the treatment reported. Enzyme inactivation, nutrient retention, and leaching should be measured directly under specified time, temperature, and water-to-leaf conditions.

Sequential treatments such as blanching followed by drying are author-proposed hypotheses. They may combine preservation and debittering mechanisms, but no included study tested an optimised sequence in *V. amygdalina*. Such combinations require factorial or response-surface experiments that measure nutrient retention, anti-nutritional factors, bitterness, microbial safety, energy use and yield.

### 4.6. Summary of Processing Effects by Evidence Class

Table 3 and Table 4 separate measured species-specific observations from proposed effects. Table 3 now reports each primary study and treatment separately and does not present cross-study percentage ranges. Table 4 contains only Class V hypotheses; durations and comparative magnitudes are excluded unless directly supported. This structure prevents heterogeneous or unmeasured outcomes from appearing quantitatively comparable.

**Table 3:** Study-specific processing evidence for *V. amygdalina*. Treatments and findings are not directly comparable across rows because processing conditions, analytical outcomes, and moisture bases differ.

Source	Treatment Actually Studied	Measured Outcomes Relevant to This Review	Defensible Interpretation	Important Limitation
[15]	Overnight soaking; blanching; abrasion with and without salt	Proximate composition, selected minerals, tannin, phytate and antioxidant measurements	Processing changed measured composition; effects apply only to the reported treatment	Treatments cannot be relabelled as standard 15- or 30-min boiling; cross-study ranges are not justified
[14]	Solar, sun and oven drying	Proximate and mineral composition	Drying changed reported concentrations	Moisture removal complicates fresh-versus-dried comparison; the study does not establish vitamin, carotenoid, phytate or oxalate retention ranges
[16]	Direct boiling and debittering before boiling	Phenolic composition, dietary fibre and related antioxidant/animal outcomes	Culinary treatment altered the outcomes actually measured	Does not support universal vitamin C, β-carotene, phytate, oxalate or trained-sensory bitterness percentages
[18]	Mechanical abrasion with salt and without salt	Proximate composition, vitamins, minerals and anti-nutritional factors	Aqueous mechanical debittering reduced several measured constituents	Not a controlled boiling, sun-drying or oven-drying experiment and cannot support those rows

Notes: Directional statements apply only to the treatment and outcomes reported by the cited study. No pooled ranges or standard 15- or 30-min boiling effects are claimed. Apparent concentration changes after drying may reflect moisture removal. Readers should consult the original reports before using any value quantitatively; the evidence is unsuitable for dietary-intake modelling or product formulation.

**Table 4:** Author-proposed processing hypotheses for *V. amygdalina* requiring species-specific testing (Class V evidence).

Proposed Treatment	Outcome Requiring Measurement	Hypothesis Only	Basis	Species-Specific Test Required
Lactic acid fermentation	Phytate and other inhibitors	May decrease; direction and magnitude unknown	General African plant-food fermentation literature [19]	Time-course using defined starter cultures and fully characterised bitter-leaf substrate
Lactic acid fermentation	Phenolic profile and bioaccessibility	May change; benefit cannot be assumed	General microbial enzyme mechanisms [19,20]	Phenolic profiling before and after fermentation followed by standardised in vitro digestion [21]
Lactic acid fermentation	Bitterness and acceptability	May change; improvement not demonstrated	General fermentation rationale only	Instrumental or trained-panel bitterness measurement and product-specific consumer testing
Lactic acid fermentation	Microbial growth, safety and endpoint	Unknown for bitter leaf	Antibacterial or antifungal activity reported for some bitter-leaf preparations or isolated compounds [1]; effects on fermentation cultures remain unknown	Starter-culture screening, pathogen control, pH/acidification kinetics and shelf-life testing
Household boiling	Net mineral delivery	Inhibitor reduction and mineral leaching may act in opposite directions	Mineral-absorption and inhibitor mechanisms [22,23,24]; mineral leaching is a mechanistic inference requiring direct measurement	Matched analysis of raw leaves, consumed cooked portion and discarded water on a common moisture basis
Blanching followed by drying	Combined retention, safety and sensory response	No advantage established over a single treatment	Author-proposed combination	Factorial optimisation with specified time, temperature, water use and drying conditions
Freeze-drying	Retention and feasibility	Potential retention and cost advantages are unquantified for this use	Laboratory-processing rationale; contextual review [1]	Species-specific comparator study including nutrient retention, stability, energy and cost

**Figure 1:**
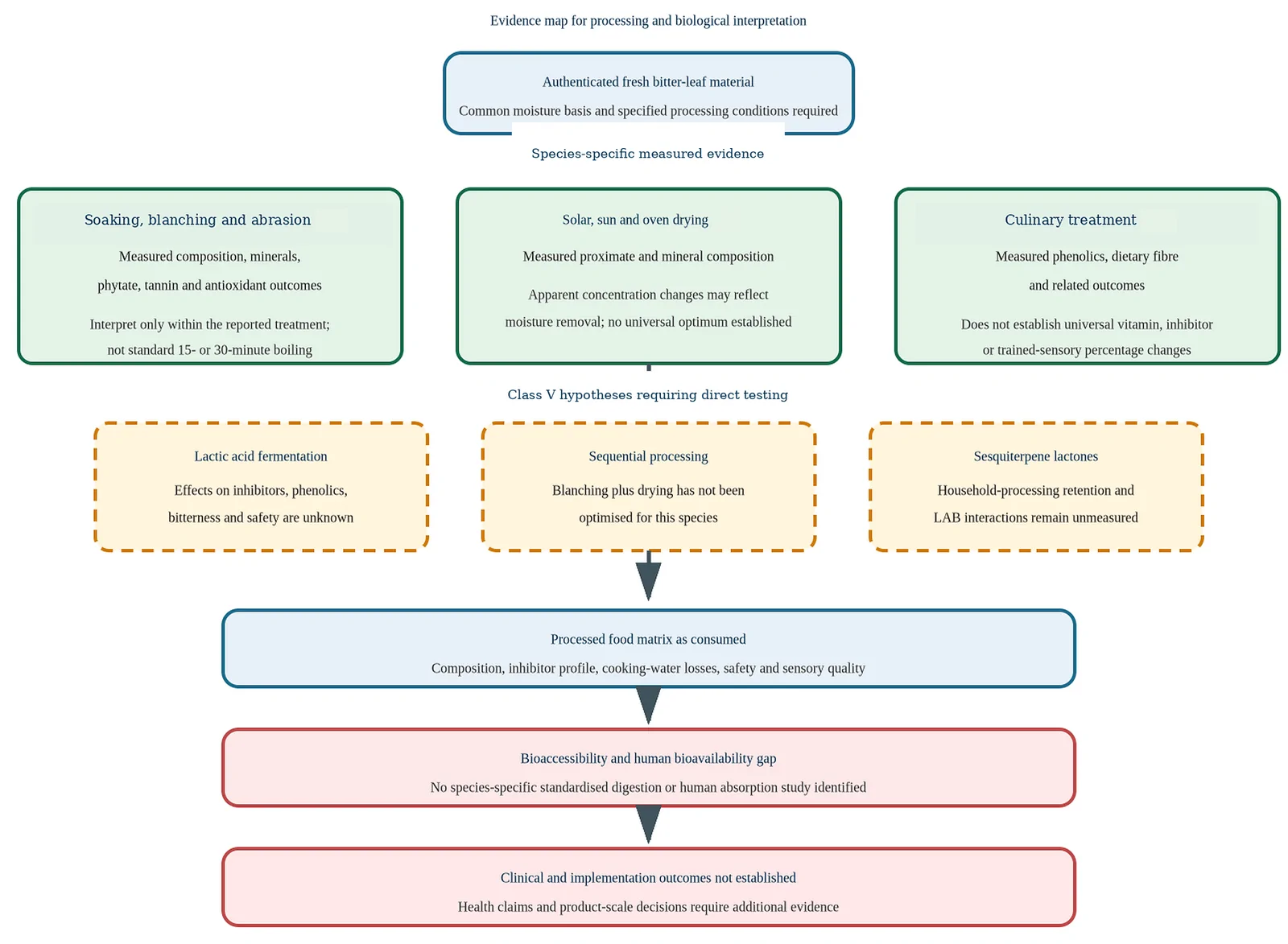
Corrected evidence map for processing and biol*ogi*cal interpretation of *Vernonia amygdalina*. Green boxes summarise outcomes measured in the cited species-specific studies; amber dashed boxes are Class V hypotheses requiring direct testing; red boxes identify unresolved bioaccessibility, human bioavailability and clinical-evidence gaps. The solid downward arrows indicate the proposed progression from measured processing evidence to research hypotheses and unresolved evidence gaps; they do not imply causality. Numeric citations formerly shown inside the image are cited here [14,15,16]. No percentage in the figure should be inferred across heterogeneous treatments.

No entry in Table 4 represents a demonstrated effect in *V. amygdalina*. The table deliberately avoids fixed fermentation times, numerical magnitudes and claims of superiority because these have not been established for this species. The relationship between measured processing outcomes, proposed hypotheses and unresolved bioaccessibility and clinical evidence gaps is summarised in Figure 1.

## 5. Mineral and Phytochemical Bioavailability from Processed Bitter Leaf

No human study identified in this review directly measured absorption or systemic bioavailability of minerals or phytochemicals from *V. amygdalina*. Evidence in this section is limited to composition, anti-nutritional factors, phytate-to-mineral ratios, and general mechanistic literature. These measures can suggest constraints on absorption but cannot quantify human bioavailability. Animal studies may investigate in vivo bioavailability, whereas establishing human bioavailability requires appropriately designed human studies. Accordingly, all indirect estimates are labelled as predicted absorption, bioaccessibility or mechanistic expectation.

### 5.1. Iron: Composition, Molar Ratios and Predicted Absorption

*Vernonia amygdalina* contains non-haem iron, but iron concentration alone does not establish absorption [25]. Phytate-to-iron molar ratios may be used as screening indicators of relative iron availability [22,26], but they are not direct measurements of absorption or validated individual-level predictions. The available processing studies use heterogeneous treatments and reporting bases, and no included *V. amygdalina* study establishes how a ‘low’ to ‘moderate’ absorption category changes after boiling. No human stable-isotope or balance study has directly measured iron absorption from bitter leaf.

### 5.2. Zinc and Calcium: Molar Ratios and Predicted Absorption

Phytate can constrain zinc absorption, and oxalate may reduce calcium availability [24]. Processing-related changes in these inhibitors can inform hypotheses about predicted absorption, but the ratios do not measure physiol*ogi*cal uptake. Fermentation-related improvements remain extrapolated from other foods, and mineral leaching may offset reductions in inhibitors. Human zinc and calcium bioavailability from processed *V. amygdalina* remains unknown.

### 5.3. Polyphenol and Phytochemical Bioaccessibility

No published in vitro digestion study identified here quantified polyphenol bioaccessibility in *V. amygdalina*. The INFOGEST consensus method is cited as a suitable standardised protocol for future work, not as evidence about bitter leaf [21]. General polyphenol literature suggests that processing and microbial metabolism can alter release and transformation [20], but isolated compounds and other plant matrices cannot establish effects within a bitter-leaf meal. The amount and identity of compounds or metabolites reaching systemic circulation therefore remain unknown. Table 5 summarises the available microbiome and colonic-fermentation evidence and its interpretive limits.

### 5.4. Leaching: Why Better Molar Ratios Do Not Necessarily Mean Better Absorption

Although boiling reduces phytate and oxalate, improving phytate-to-mineral molar ratios, it also causes minerals to leach into the cooking water, which is often discarded. Therefore, improved molar ratios do not necessarily translate into greater mineral absorption. The amount of iron, zinc, or calcium ultimately absorbed depends on the balance between the reduction in anti-nutritional factors and the loss of minerals during cooking. No study has measured both processes simultaneously in *V. amygdalina*. Consequently, it is more accurate to state that boiling improves the inhibitor profile of the consumed leaves and is predicted to enhance mineral absorption, but its effect on the actual amount of minerals absorbed remains unknown. Future studies should analyse both the cooked leaves and the discarded cooking water to determine the true nutritional impact of processing. No study has yet reported quantitative mineral-loss values for the household cooking of *V. amygdalina*; this absence of species-specific leaching data is a critical research gap that currently prevents any net estimate of mineral bioavailability from cooked bitter leaf.

## 6. Gut Microbiome Interactions with *Vernonia amygdalina* Fractions

### 6.1. Dietary Fibre, Gut Microbiota and Short-Chain Fatty Acid Production

*Vernonia amygdalina* contains dietary fibre, but its fermentation by human gut microbiota has not been demonstrated in a species-specific colonic model. General non-starch-polysaccharide literature provides a rationale for investigating SCFA production and changes in microbial composition [27]; it does not establish selective stimulation of *Lactobacillus* or *Bifidobacterium* by bitter leaf. The appropriate conclusion is that bitter-leaf fibre has hypothesised, not demonstrated, prebiotic potential. Species-specific in vitro colonic fermentation and controlled human feeding studies are required.

### 6.2. Polyphenol Biotransformation by Gut Microbiota

Polyphenols that escape small-intestinal absorption can be transformed by gut microorganisms [20]. Quercetin metabolism has been studied primarily as an isolated compound or in matrices other than *V. amygdalina*. The bitter-leaf matrix may alter release, microbial transformation and absorption, so isolated-quercetin findings cannot be transferred directly to a bitter-leaf meal. No human study has established the systemic bioavailability of bitter-leaf-derived quercetin metabolites. Future studies should combine a standardised meal, plasma and urinary metabolite analysis, and microbiome profiling.

## 7. Food Product Development: From Raw Ingredient to Scalable Product

### 7.1. Translating Research into Food Products

No published evidence was identified of a compositionally standardised *V. amygdalina* food product with validated shelf life, consumer acceptance and widespread formal-market adoption. This statement applies to the academic and grey literature searched and does not constitute a commercial-market census. Informal sale of fresh or dried leaves should not be conflated with validated product readiness. Product development is constrained by bitterness, variable raw material, processing consistency, safety assurance, packaging, market evidence and regulatory classification.

### 7.2. Promising Product Formats

The formats below are conceptual options proposed by the authors, not experimentally validated products or formal technology-readiness determinations. They are retained to organise research questions rather than to imply technol*ogi*cal feasibility, consumer acceptance or commercial readiness. Evidence should progress from ingredient characterisation and safe-process development to prototype testing, shelf-life assessment, sensory and consumer studies, regulatory classification and, where relevant, human research.

1. Fermented leaf paste: an author-proposed prototype requiring species-specific starter-culture screening, fermentation kinetics, food-safety validation, shelf-life testing and sensory evaluation. No claim of improved bitterness, nutrient retention or cultural acceptability is established for this product.
2. Standardised leaf powder: a potential ingredient requiring defined raw-material specifications, validated drying conditions, nutrient and contaminant testing, oxidative-stability data, packaging studies and product-specific acceptability testing. Microencapsulation remains a separate untested concept.
3. Bitter-leaf-enriched fermented cereal food: a proposed prototype for testing nutrient composition, inhibitor levels, safety and acceptance. Evidence that cereal fermentation reduces phytate cannot establish improved mineral absorption after adding bitter leaf, and suitability for children or school feeding has not been demonstrated.
4. Dehydrated leaf flakes: a proposed convenience format requiring a species-specific blanching/drying protocol, shelf-life validation and sensory testing. Results from *Moringa oleifera* products cannot be assumed to apply to the more bitter *V. amygdalina* matrix.
5. Standardised extract capsule or tablet: this would be a health product rather than a conventional food application and may fall within medicines or complementary-medicines regulation depending on composition, dose and claims. Its development would require product-specific safety, quality, efficacy and regulatory assessment.

These concepts identify potential research pathways only. No statement in this section should be interpreted as evidence of demonstrated commercial feasibility, consumer acceptance, public-procurement suitability or health benefit.

### 7.3. Consumer Acceptance and Behaviour Change

Consumer evidence specific to *V. amygdalina* products is limited; much of the available literature concerns indigenous leafy vegetables as a broader category [28,29]. Species, preparation, bitterness, familiarity, age, setting, and price may produce different responses. General findings about younger consumers, rural identity, purchase intention, or nutritional messaging should therefore be treated as contextual hypotheses rather than bitter-leaf-specific conclusions. Product-specific studies should evaluate sensory acceptance, actual willingness to purchase, repeat use and affordability in defined populations. Table 6 outlines the author-proposed product-development stages and the evidence required at each decision gate.

**Table 6:** Author-proposed development stages for potential *V. amygdalina* products. Categories are qualitative research stages, not validated technology-readiness ratings or forecasts.

Product Format	Target Consumer	Current Evidence Stage	Key Barrier	Priority Research Action	Next Decision Gate
Fermented leaf paste	Rural households; traditional cooks	Concept only	Starter culture standardisation; shelf-life under ambient conditions; unknown fermentation kinetics	Characterise fermentation of bitter leaf substrate; starter culture development; shelf-life study	Demonstrate controlled fermentation, safety, stability and acceptable sensory profile
Standardised oven-dried leaf powder	Food processors; school nutrition programmes	Ingredient-characterisation stage	Anti-nutritional factor residues; bitterness at nutritional dose; oxidative stability	Optimise drying temperature and time; evaluate microencapsulation; shelf-life and sensory studies	Validate drying, specifications, stability, safety and use in a defined food
Microencapsulated powder (groundnut oil matrix)	Urban consumers; commercial fortification	Concept only	No published study for this species; capital cost of encapsulation	Proof-of-concept study; bioaccessibility comparison against unencapsulated powder	Demonstrate formulation feasibility and compare bioaccessibility with unencapsulated powder
Bitter leaf-enriched fermented porridge (*mageu*, *ogi*)	Children; school nutrition; maternal health	Concept only	Child acceptance of residual bitterness; unknown LAB interaction with bitter leaf fractions	Acceptability study in school-age children; optimise inclusion rate for acceptability–nutrition balance	Demonstrate formulation, safety, nutrient profile and target-population acceptance
Dehydrated leaf flakes for soup	Peri-urban and urban consumers	Analogue-informed concept	Standardised quality specifications; regulatory framework for ILV products	Blanching and drying optimisation; HACCP protocol development; regulatory dossier preparation	Develop and validate a bitter-leaf-specific blanching/drying and HACCP process
Standardised extract capsules or tablets	Health-conscious urban consumers; trial participants	Preclinical product-definition stage	Drug–nutrient interaction uncertainty; no Phase I safety data; no applicable registration discipline (Section 8.1)	Phase I dose-escalation trial; pharmacokinetic study; regulatory engagement with SAHPRA	Confirm regulatory category, standardise product and establish safety before any justified human study

**Figure 2:**
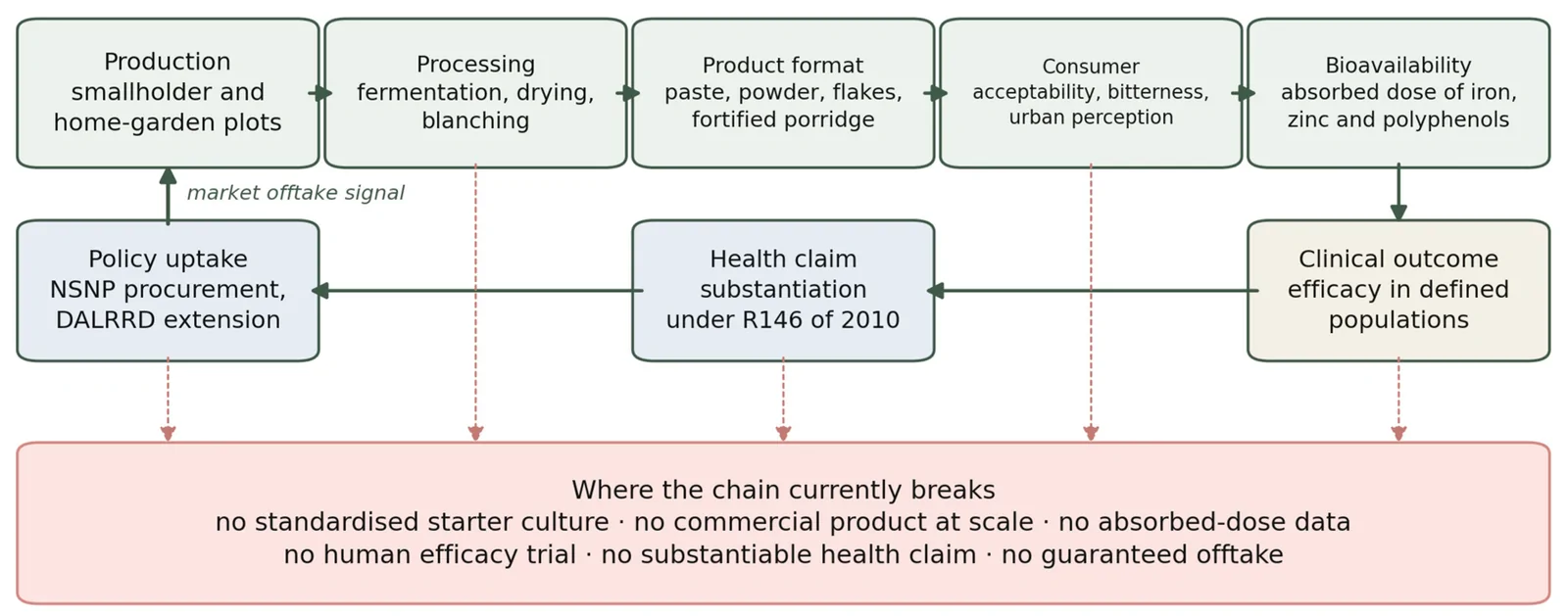
Conceptual framework for integrating *Vernonia amygdalina* into the food system. The framework illustrates the proposed pathway from production and processing to product development, consumer acceptance, bioavailability, clinical outcomes, health claims, and policy adoption. Policy support can, in turn, promote market demand and encourage production. This framework is conceptual and has not been empirically validated. Solid arrows show proposed directions, dashed arrows mark evidence gaps, and box colours distinguish development, policy, clinical and evidence-gap stages; none implies causality.

HACCP = Hazard Analysis and Critical Control Points; LAB = lactic acid bacteria; SAHPRA = South African Health Products Regulatory Authority. The stages indicate the next evidence needed and should not be interpreted as predictions of time to commercial scale. The proposed pathway linking production, processing, product development, consumer acceptance, bioavailability, clinical outcomes, health-claim substantiation and policy uptake is illustrated in Figure 2.

## 8. Regulatory and Policy Considerations

### 8.1. Food Law and Health Claims

Regulatory treatment depends on whether a product is marketed as a conventional food, health supplement, complementary medicine or other medicine, as well as its composition, dose, presentation and claims. Conventional foods fall under the Foodstuffs, Cosmetics and Disinfectants Act and applicable labelling regulations [30,31,32]. A nutrient-content claim requires compliance with the applicable compositional and labelling criteria; therapeutic or disease-related claims cannot be inferred from food-composition evidence. Because the 2023 labelling instrument cited here was published as a draft, developers must verify the final legal position and any amendments at the time of submission or marketing. This review does not provide legal advice or conclude that every non-nutrient claim is categorically prohibited.

Comparable distinctions apply in Nigeria, Ghana and Kenya, but the cited statutes establish broad regulatory authority rather than product-specific approval for *V. amygdalina* [33,34,35,36]. The manuscript therefore does not claim that bitter leaf is absent from every national register or that the same evidence package applies in each jurisdiction. Current product classification and registration requirements should be confirmed directly with the responsible authority before commercialisation.

### 8.2. Medicines Law and the Complementary Medicines Route

An extract presented for therapeutic use may be regulated under the Medicines and Related Substances Act and its regulations rather than as a conventional food [37,38]. SAHPRA distinguishes low-risk complementary-medicine or health-supplement claims from higher-risk therapeutic claims and applies product-specific requirements for quality, safety and efficacy [39,40,41]. Traditional evidence may be relevant to some low-risk indications, whereas stronger clinical evidence is required for higher-risk claims. The applicable pathway depends on the actual formulation, dose, dosage form and proposed claim. The European traditional herbal medicinal product framework provides a comparative example but does not govern South African classification [42].

### 8.3. Bioprospecting, Benefit-Sharing and Indigenous Knowledge

The commercial development of *V. amygdalina* may fall within South Africa’s biodiversity and indigenous knowledge legislation [43,44,45]. Depending on whether the proposed activity constitutes bioprospecting, biotrade, collection or export involving indigenous biological resources, relevant permits, notifications, material-transfer agreements or benefit-sharing arrangements may be required. Applicability should therefore be confirmed with the responsible authority before commercialisation.

### 8.4. Policy Integration Opportunities

Potential policy applications, including public nutrition programmes, remain proposals. Before procurement can be considered, a product would require standardised composition, safety and quality assurance, reliable supply, acceptable sensory performance, affordability and compliance with the relevant procurement and food regulations. No claim is made that current evidence is sufficient for school-feeding implementation.

## 9. Proposed Framework for Clinical Research

**Figure 3:**
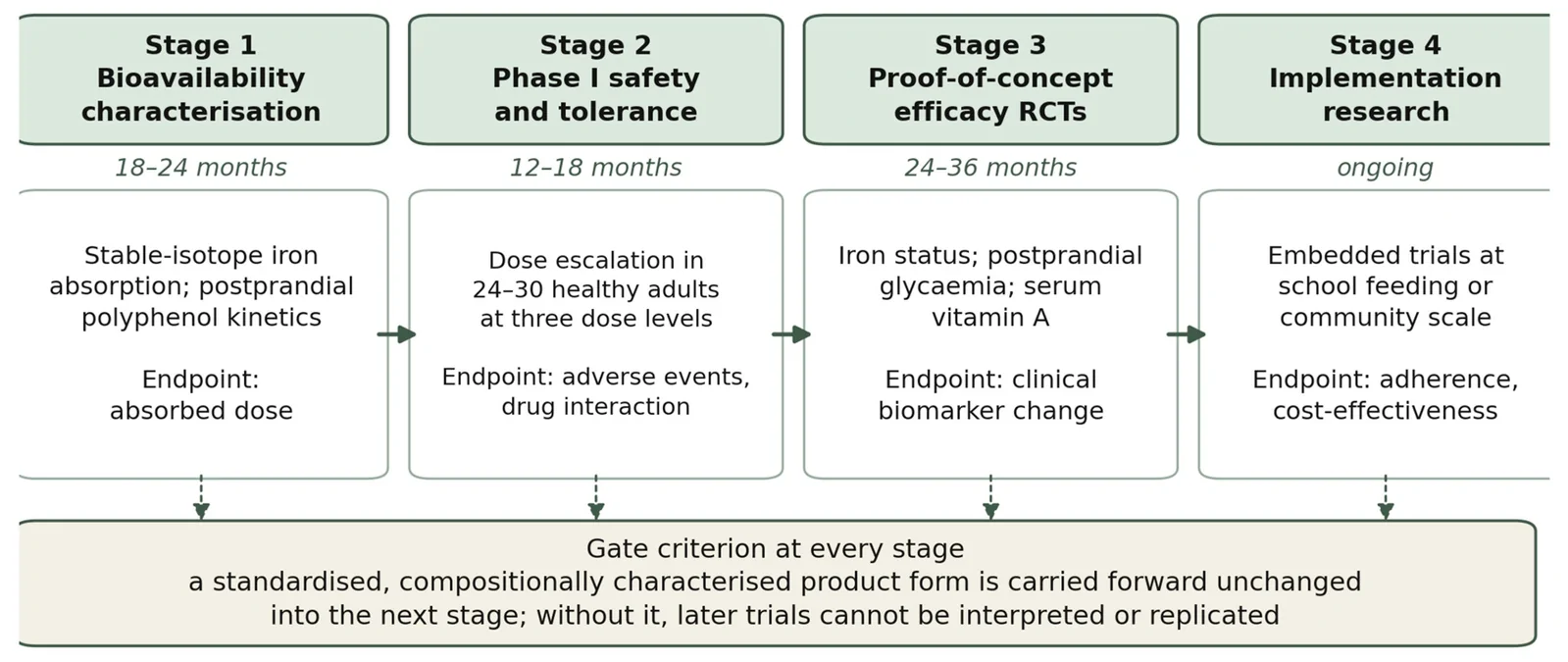
Author-proposed and unvalidated research framework for *V. amygdalina* products. The sequence is a planning hypothesis, not an established methodol*ogi*cal or regulatory requirement. Product identity, composition, and safety precede in vitro bioaccessibility and any justified human tolerability, absorption, or efficacy study; solid horizontal arrows indicate progression between stages, while dashed downward arrows indicate that each stage must satisfy the gate criterion before progression. Implementation research is conditional on preceding evidence.

The framework in Figure 3 was developed by the authors as a research-planning concept; it is not an established or regulator-endorsed clinical-development pathway. Research should begin with botanical authentication, product standardisation, compositional and contaminant analysis, process validation and fit-for-purpose safety assessment. Standardised in vitro digestion may then estimate bioaccessibility [21]. If a product and intended claim justify human research, initial studies should establish tolerability and measured absorption using an appropriate food-study design, followed by adequately powered efficacy trials. “Phase I” terminology is avoided unless the product is formally investigated as a medicine. Priority endpoints are iron status, vitamin A status and glycaemic control, but their inclusion must follow biol*ogi*cal plausibility, product composition, public-health relevance, feasibility and ethical review. Implementation and cost-effectiveness research would be appropriate only after safety, acceptability and efficacy are demonstrated.

## 10. Critical Synthesis: Strengths, Limitations and Research Priorities

### 10.1. What Evidence Supports

Current evidence supports describing *V. amygdalina* as a nutrient-containing indigenous leafy vegetable and shows that processing can change measured composition [1,3,14,15,16,18,25]. It does not support a single quantitative effect for household boiling or drying across heterogeneous studies. Reductions in anti-nutritional factors may improve inhibitor profiles, but neither molar ratios nor composition establish mineral absorption in humans.

### 10.2. What Evidence Does Not Support

Despite promising laboratory and animal studies, there is currently no human evidence demonstrating the therapeutic benefits of *V. amygdalina*. The review identifies four major knowledge gaps. (1) No species-specific studies have investigated the effects of fermentation. (2) All estimates of mineral absorption are based on predictive models rather than direct human measurements. (3) Improved phytate-to-mineral ratios do not necessarily indicate greater mineral absorption because minerals may also be lost during cooking. (4) The combined effects of processing methods, meal composition, and individual gut microbiota on nutrient absorption remain unknown. In addition, no standardized commercial bitter leaf products with validated nutritional composition and clinical evidence have been reported**.**

### 10.3. Limitations of This Review

This structured narrative review was not protocol-registered, did not preserve database-specific search dates or auditable screening-flow counts, used one primary screener, and did not apply a formal risk-of-bias tool. These limitations increase the possibility of selection bias and missed studies. Heterogeneity in treatments, moisture basis, plant source, and analytical procedures prevented pooled quantitative comparison. The product and clinical frameworks are author-generated proposals. The market assessment relied on academic and grey literature rather than a formal commercial database search, and the regulatory discussion is time-sensitive and must be checked against current official requirements.

## 11. Conclusions

*Vernonia amygdalina* is a nutrient-rich indigenous leafy vegetable whose composition is altered by processing. The present evidence does not establish universal percentage losses during boiling or drying, human nutrient bioavailability, microbiome effects, clinical benefits, or commercially validated food products. Fermentation represents a promising research opportunity, but species-specific studies are required before its nutritional, sensory or technol*ogi*cal advantages over other processing methods can be established. Future work should use authenticated and standardised material, fully specified processing conditions, common moisture bases, validated in vitro digestion, product-specific safety and sensory testing, and appropriately designed human studies where justified. The proposed food formats and research sequence are planning concepts rather than established interventions. Evidence from such studies is required before bitter-leaf products can be promoted for specific nutritional or public-health outcomes.

## Figures and Tables

**Table 1:** Evidence classification framework applied throughout this review.

Class	Type of Evidence	Species	Permitted Interpretive Use
I	Human intervention study (randomised or controlled)	*V. amygdalina*	May support causal inference for the studied intervention and outcome when design and execution are adequate; none identified for the review endpoints.
II	Human observational study	*V. amygdalina*	May support association in the studied population; residual confounding remains possible; none identified for absorption or clinical endpoints.
III	Animal or ex vivo study	*V. amygdalina*	Supports biol*ogi*cal plausibility in the tested model; does not establish human effects or dietary-dose relevance.
IVa/IVb	In vitro study on *V. amygdalina* (compositional analysis, processing experiment, simulated digestion or colonic fermentation). IVa = quantitatively measured effect; IVb = qualitative, directional or uncertain observation.	*V. amygdalina*	Supports only the species-specific measured compositional, processing or in vitro outcome. IVa denotes quantitative measurement; IVb denotes qualitative or uncertain direction.
V	Extrapolation from other species, prediction from a general dietary model, or mechanistic inference from chemistry	Other or none	Generates a hypothesis or contextual expectation only; does not support a quantitative or species-specific claim.

**Table 2:** Study-level characteristics and interpretive limits of sources used in the processing synthesis. The cited sources [1,13,14,15,16,17,18,19] include species-specific primary studies and contextual methodological, review, or fermentation evidence.

Source	Material and Plant Part	Processing Examined	Parameters Reported	Cultivar Authentication	Moisture Basis Stated	Processing Conditions Fully Specified	Analytical Method Identified	Evidence Class
[15]	*V. amygdalina* leaves	Overnight soaking; blanching; abrasion with salt; abrasion without salt	Proximate composition; minerals; tannin; phytate; antioxidant measurements	Species reported; cultivar not reported	Varied by parameter	Partly	Reported	IVa
[14]	*V. amygdalina* leaves	Solar, sun and oven drying	Proximate and mineral composition	Species reported; cultivar not reported	Fresh/dried comparison reported; interpretation affected by moisture loss	Partly	Reported	IVa
[16]	*V. amygdalina* leaves	Direct boiling and debittering before boiling	Phenolic composition; dietary fibre; antioxidant-related and animal outcomes	Species reported; cultivar not reported	Not directly comparable with nutrient-retention studies	Partly	Reported	IVa for measured outcomes; III for animal outcomes
[18]	*V. amygdalina* leaves	Mechanical abrasion with salt and without salt; not a boiling or drying study	Proximate composition; vitamins; minerals; phytate; tannin; oxalate; cyanide	Species authenticated; cultivar not reported	Mixed wet- and dry-weight reporting	Yes for the abrasion treatments	Reported	IVa
[1]	Review of *V. amygdalina* literature	Secondary synthesis; no single processing protocol	Composition, phytochemistry, agronomy and pharmacology	Not applicable	Varies	Not applicable	Varies	V when used for context
[19]	African fermented plant foods; not *V. amygdalina*	General lactic acid fermentation	General fermentation mechanisms and outcomes	Not applicable	Varies	Varies	Varies	V for bitter-leaf inference

“Partially” indicates that the parameter was stated for some but not all treatments or constituents. Evidence class is as defined in Table 1. Entries in this table describe the reporting completeness of each source, not its scientific quality. Within Class IV, quantitatively measured effects are designated Class IVa and qualitative, directional or otherwise uncertain observations are designated Class IVb.

**Table 5:** Gut microbiome and colonic fermentation evidence for *V. amygdalina*, classified by study type.

Observation	Model or Study Type	Species Studied	Evidence Class	What Would Establish Human Relevance
Potential fermentability of bitter-leaf dietary fibre	Extrapolated from dietary-fibre fermentation principles; not demonstrated for *V. amygdalina* [27]	Not species-specific	V	A species-specific in vitro colonic-fermentation study using standardised *V. amygdalina* leaf material
Possible change in *Lactobacillus* or *Bifidobacterium* abundance	Extrapolated from dietary-fibre fermentation principles [27]	Not species-specific	V	Controlled human feeding study with 16S or shotgun sequencing at baseline and after defined intake
Possible SCFA production	Extrapolated from dietary-fibre fermentation principles [27]	Not species-specific	V	Faecal and, ideally, plasma SCFA measurement in a human intervention
Butyrate supports colonocyte energetics, mucosal integrity and anti-inflammatory signalling	Established mechanistic and human literature [27]	Not species-specific	IV/V	Not required for the mechanism; required for attribution of any such effect to bitter leaf intake
Possible regional differences in colonic fermentation	Mechanistic inference from in vitro SCFA ratios	Not measured	V	Regional sampling or stable-isotope tracing; not feasible at present
Possible metabolic effects of microbially generated SCFAs	Mechanistic inference	Not measured	V	Human intervention with metabolic endpoints and portal or peripheral propionate kinetics
Possible conversion of quercetin to phenolic-acid metabolites	Established pathway in isolated-compound studies [20]	Not bitter leaf matrix	V	Postprandial plasma and urinary metabolite profiling after a standardised bitter leaf meal
Inter-individual variation in metabolite yield according to microbiome composition	Mechanistic inference from polyphenol literature [20]	Not measured	V	Microbiome-stratified pharmacokinetic study

SCFA = short-chain fatty acid. No Class I, II, or III evidence relating *V. amygdalina* to microbiome outcomes was identified.

## Data Availability

No new data were created or analyzed in this study. Data sharing is not applicable to this article.

## Appendix A

**Table A1:** Representative electronic search syntax. Core concept blocks were adapted to each database. Because searches were iterative and database-specific execution dates and flow counts were not preserved, the table documents the approach but should not be interpreted as a fully reproducible systematic-review search log.

Database/Source	Representative Search String
Scopus	TITLE-ABS-KEY ((“*Vernonia amygdalina*” OR “bitter leaf” OR ewuro OR onugbu OR omululuza) AND (process* OR boil* OR blanch* OR dry* OR ferment* OR cook* OR “nutrient composition” OR antinutrient* OR phytate OR oxalate OR bioavailab* OR bioaccess* OR “gut microbiota” OR “product development” OR sensory OR policy OR regulat*))
Web of Science Core Collection	TS = ((“*Vernonia amygdalina*” OR “bitter leaf”) AND (process* OR boil* OR blanch* OR dry* OR ferment* OR antinutrient* OR phytate OR oxalate OR bioavailab* OR bioaccess* OR “gut microbi*” OR “food product*” OR sensory OR regulat*))
PubMed	(“*Vernonia amygdalina*” [Title/Abstract] OR “bitter leaf” [Title/Abstract]) AND (processing [Title/Abstract] OR boiling [Title/Abstract] OR blanching [Title/Abstract] OR drying [Title/Abstract] OR fermentation [Title/Abstract] OR antinutrient* [Title/Abstract] OR phytate [Title/Abstract] OR oxalate [Title/Abstract] OR bioavailability [Title/Abstract] OR bioaccessibility [Title/Abstract] OR “gut microbiota” [Title/Abstract])
ScienceDirect	(“*Vernonia amygdalina*” OR “bitter leaf”) AND (processing OR boiling OR blanching OR drying OR fermentation OR antinutrients OR bioavailability OR bioaccessibility OR “gut microbiota”)
Google Scholar	“*Vernonia amygdalina*” (processing OR boiling OR drying OR fermentation OR bioavailability OR bioaccessibility OR antinutritional); first 200 results considered
AJOL and Sabinet (regional)	(“*Vernonia amygdalina*” OR “bitter leaf” OR muxe OR murungurwa) AND (processing OR nutrition OR bioavailability OR policy OR regulation)
Old literature/official sources	FAO, WHO, HSRC, South African government and regulatory websites; targeted searches for policy, labelling, medicines and indigenous-knowledge instruments

Note: * is a truncation wildcard used to retrieve variant word endings.
